# Phylogenetic Analysis, Pulse-Amplitude-Modulated (PAM) Fluorometry Measuring Parameter Optimization, and Cell Wall Disintegration of *Chlorella vulgaris* K-01

**DOI:** 10.3390/microorganisms13040711

**Published:** 2025-03-21

**Authors:** Zhenyu Zhang, Xiaoli Zhang, Yinqiang Wu, Li-Hua Yao, Pengcheng Fu

**Affiliations:** 1 School of Life Science, Jiangxi Science & Technology Normal University, Nanchang 330013, China; 2 State Key Laboratory of Marine Resource Utilization in South China Sea, Hainan University, Haikou 570228, China

**Keywords:** *Chlorella*, PAM fluorometry, cell lysis, prebiotics, high-pressure homogenization, ultrasonication

## Abstract

*Chlorella* is a rich source of nutrients. In addition to its nutritional value, it exhibits versatile biological activities. New strains have been extensively identified and investigated in recent years to expand the potential of *Chlorella*. The accurate measurement of pulse-amplitude-modulated (PAM) fluorometry parameters and effective microalgal cell lysis are foundational for advanced studies of novel *Chlorella* species. In this study, ribosomal small subunit (SSU)-internal transcribed spacer (ITS) phylogenetic analysis and internal transcribed spacer 2 (ITS2) secondary structure analysis were employed to identify a new *Chlorella* species. The dark adaptation time, the duration of the saturation pulse, the intensity of actinic light, and the duration of actinic light exposure for PAM fluorometry measurements were optimized. Different conditions of ultrasonication and high-pressure homogenization (HPH) for microalgal cell lysis were compared. *Chlorella vulgaris* K-01 was identified. The suitable duration for dark adaptation, the saturation pulse, and the actinic light were 15 min, 200 milliseconds, and 30 s, respectively. The suitable intensity of actinic light was 191 μE/(m^2^·s). For microalgal cell lysis, HPH could achieve 98.65% cell lysis efficiency at 30 kpsi (207 MPa), whereas ultrasonication attained an efficiency of 45.47% (300 W for 30 min). These results facilitate further study on the physiology and the composition of *Chlorella vulgaris* K-01.

## 1. Introduction

Microalgae are renowned for their rich composition of biochemicals, including proteins, carotenoids, and essential fatty acids, holding significant potential for human health and nutrition [1]. Their rapid growth capabilities, combined with high production efficiency and nutrient density, position microalgae as superior candidates for the large-scale cultivation of bioproducts [2,3]. The practice of incorporating microalgae into human diets extends back millennia, initially as a means to endure periods of famine [4]. Over time, the nutritional benefits of these microorganisms have become widely acknowledged [5]. In contemporary times, specific microalgae such as *Spirulina (Arthrospira)*, *Chlorella*, and *Haematococcus* have been extensively cultivated and marketed as dietary supplements [1,6,7]. They are now supplemented into a variety of food products, ranging from noodles and chewing gum to beer, tea, bread, and soft drinks, reflecting their versatility and nutritional value [8,9,10,11,12].

First identified in 1890 by the distinguished microbiologist Dr. Martinus Willem Beijerinck, *Chlorella* has emerged as a microalgal species of significant interest due to its nutritional properties and resilience to adverse environmental conditions [13,14]. *Chlorella* stands out for its wealth of nutrients and bioactive compounds, which have the potential to improve human health and prevent a variety of diseases [15]. These attributes make *Chlorella* a promising natural alternative to synthetic compounds and pharmaceuticals. The variability in the levels and types of these bioactive compounds within *Chlorella*, influenced by cultivation conditions and specific species, has prompted ongoing research into the isolation and exploration of new *Chlorella* strains [16,17]. With advancements in molecular identification techniques [18,19], the understanding of *Chlorella* has significantly deepened with the discovery of over 20 species and more than 100 strains of *Chlorella* (https://www.algaebase.org/browse/taxonomy/#6964, accessed on 1 March 2025) [20,21]. Four subordinate taxa were widely studied: *Chlorella vulgaris* Beijerinck [13], *Chlorella lobophora* Andreyeva [22,23], *Chlorella variabilis* Shihira and Krauss [20,24], and *Chlorella sorokiniana* Shihira and Krauss [23,25]. In recent years, research has shed light on the potential psychoactive effects of *Chlorella* biomass, which include mood regulation, the enhancement of memory and learning capabilities, and stress alleviation through the modulation of brain c-fos expression and the hypothalamic–pituitary–adrenal axis [26,27]. Furthermore, *Chlorella* has demonstrated significant effects on the gut microbiota of both humans and animals [28,29,30]. Cell content from *Chlorella* biomass such as polysaccharides could modulate gut microbiota [31], positioning it as a potential source of prebiotics [32]. Despite the increasing interest in uncovering new species and biological activities within the *Chlorella* genus, two primary challenges impede the full utilization of *Chlorella* biomass: monitoring the physiological status and achieving efficient microalgal cell lysis [33,34]. The composition and nutritional value of *Chlorella* biomass are heavily influenced by cultivation conditions and the physiological state of the species [35,36,37]. Additionally, the cell wall content varies among *Chlorella* species, leading to species-specific requirements for cell content extraction [34,38,39]. To assess the physiological status of *Chlorella*, pulse-amplitude-modulated (PAM) fluorometry stands out as the most widely used, real-time, and non-invasive method [40,41,42]. It measures photosynthesis efficiency and chlorophyll fluorescence, both of which are indicative of the microalgae’s physiological health [43]. The measurement conditions for PAM fluorometry were empirical, leading to potentially inaccurate results [44]. For cell lysis, techniques such as high-pressure homogenization and ultrasonication are commonly employed. These methods have been widely used to break the cell walls of *Chlorella*, with reported variations in efficiency across different species [34]. Therefore, establishing precise measurement parameters for PAM fluorometry and refining the conditions for cell lysis are essential steps when working with newly discovered *Chlorella* strains. These procedures are foundational for advancing future investigative efforts and unlocking the full potential of these microalgal strains in various applications.

In previous studies, our laboratory isolated a novel chlorophyte *Chlorella vulgaris* K-01 [45]. Initial BLAST (https://blast.ncbi.nlm.nih.gov/Blast.cgi, accessed on 23 February 2021) analysis revealed the strain could be a member of *Chlorella vulgaris*, and the biomass of the strain demonstrated exceptional cadmium biosorption capacity (17.46 mg/g dry biomass) [45]. Subsequent heterotrophic cultivation experiments further revealed its robust growth kinetics and selenium bioaccumulation potential (up to 154 µg organic Se/g DW), positioning it as a promising candidate for bioremediation and nutraceutical applications [46]. However, while these studies established foundational insights into the strain’s environmental adaptability and metabolic versatility, critical taxonomic and methodological gaps remain unresolved. The original blast analysis relied on partial sequence similarity without comprehensive comparison to related taxa, leaving phylogenetic position unknown and uncertainties in identification. Furthermore, preliminary physiological assessments employed empirically adopted PAM fluorometry parameters from unrelated species, potentially compromising photosynthetic efficiency measurements. Additionally, the lack of a systematic evaluation of cell disruption methods hinders efficient extraction of intracellular components. To address these limitations, this study aims to (1) verify the identity of *Chlorella vulgaris* K-01 through phylogenetic analysis and ITS2 secondary structure modeling, (2) establish species-specific PAM fluorometry protocols, and (3) optimize cell wall disintegration strategies. These advancements could support further study on the physiology and composition of the strain.

## 2. Materials and Methods

### 2.1. Culture Condition and Yield of Biomass

KC medium was prepared following the protocol described by Osman et al. [47] and used for the cultivation of the microalga *Chlorella vulgaris* K-01. It contains KNO_3_ (0.405 g), NaCl (0.235 g), NaH_2_PO_4_·2H_2_O (0.235 g), Na_2_HPO_4_·12H_2_O (0.18 g), MgSO_4_·7H_2_O (0.125 g), CaCl_2_·7H_2_O (0.01 g), FeSO_4_·7H_2_O (3 mg), and Rippka-developed trace elements solution (1 mL) [48]. The microalgal cultures were maintained in flasks within incubators (ZCZY-CN Shanghai Zhichu Instrument Co., Ltd., Shanghai, China). The incubation parameters were optimized to a constant temperature of 28 °C, with agitation at 130 rpm to ensure uniform mixing. The cultures were exposed to a continuous light supply with a photon flux density of 50 μE/(m^2^·s) to support photosynthetic activity. Microalgal biomass was harvested via centrifugation at 5000× *g* for 15 min. Following centrifugation, the supernatant was carefully decanted and discarded.

### 2.2. Molecular Identification

The strain *Chlorella vulgaris* K-01 was initially obtained from the discharge stream of a salt concentration facility [45]. To extract the genomic DNA from this strain, a DNA extraction kit (Tiangen Biochemical Technology Co., Ltd., Beijing, China) specifically designed for plants was utilized. Following extraction, the genomic DNA was subjected to polymerase chain reaction (PCR) amplification using a set of primers: NS1–NS8 and ITS1–ITS4, as referenced in previous studies [18,49]. These primers were selected to amplify specific regions of the 18S rDNA (small subunit, SSU) and the internal transcribed spacer (ITS) regions. Amplification was performed using a Bio-Rad T100 PCR Gradient Thermal Cycler (Bio-Rad, Hercules, CA, USA) with the following protocol: initial denaturation at 94 °C for 3 min; 30 cycles of 94 °C for 30 s, 55 °C for 30 s, and 72 °C for 1 min; final extension at 72 °C for 5 min. The PCR products were analyzed and purified through agarose gel electrophoresis (DYY-8C, LIUYI BIOTECHNOLOGY Co., Ltd., Beijing, China), employing a DNA Gel Extraction Kit (Beyotime Biotech Inc, Shanghai, China), prior to sequencing on a 3730xl DNA analyzer (Thermo Fisher Scientific Inc., Waltham, MA, USA). For phylogenetic analysis, small subunit ribosomal RNA and internal transcribed spacer sequences from our strain and other closely related taxa, as identified through BLAST searches [50]. The phylogenetic analysis was performed using the MEGA 11 software suite [51], with *Parachlorella* sp. serving as the reference outgroup. Multiple alignments were performed using ClustalW (Version: 2.1) with default parameters [52]. The GTR+I+G model, which was determined to be optimal for constructing the maximum likelihood (ML) phylogenetic tree based on the Akaike information criterion from Modeltest, was selected for this analysis [53]. The robustness of the tree was evaluated by calculating bootstrap values using the ML method with 1000 iterations [54]. To discern between *Chlorella* and *Micractinium*, which are closely related in terms of genetics and morphology, the secondary structures of the ITS-2 region were predicted for both species using Mfold with default parameters [55]. Subsequently, the microalgal sequences under investigation were manually inspected and compared at the level of secondary structure to facilitate species distinction [56,57]. For comparative purposes, *Chlorella vulgaris* SAG 211-11b was used to represent the typical ITS2 structure of *Chlorella*, while *Micractinium pusillum* CCAP 248/5 was chosen to represent the typical ITS2 structure of *Micractinium*. Distinctive features between *Chlorella* and *Micractinium* were annotated based on a previous publication [56].

### 2.3. Optimization of PAM Fluorometry Parameters

Microalgal samples were prepared by diluting fresh biomass in phosphate-buffered saline (PBS) to a final cell density of 1 × 10^6^ cells/mL. The diluted samples were then used to optimize the parameters for PAM fluorometry using a Dual-PAM-100 fluorometer (Heinz Walz GmbH, Effeltrich, Germany). The optimization involved varying the dark adaptation time (5, 10, 15, 20, 25, 30, and 60 min), the duration of the saturation pulse (100, 150, 200, 250, 300, 400, 500, and 600 milliseconds), the intensity of actinic light (28, 74, 101, 140, 191, 532, and 835 μE/(m^2^·s)), and the duration of actinic light exposure (10, 20, 30, 40, 50, and 60 s). An additional cuvette kit was used for measuring PAM fluorometry parameters specific to microalgae. All experiments were conducted at room temperature without stirring [58], following the standard operating procedures provided by the manufacturer. Chlorophyll-fluorescence-related parameters were recorded through the graphical user interface of the software (DualPAM v3.18, Heinz Walz GmbH, Effeltrich, Germany) supplied by the manufacturer. ETRmax, a, and IK were calculated with the default settings of the software (https://www.walz.com/files/downloads/pan/PAN11001.pdf, accessed on 16 July 2024).

### 2.4. Microalgal Cell Wall Disruption

Fresh *Chlorella vulgaris* K-01 biomass in the logarithmic growth period was harvested and resuspended in KC medium, after rinsing twice with PBS buffer. The concentration of *Chlorella vulgaris* K-01 biomass was adjusted to 0.5 g/L. The microalgal solution was dispensed into 10 mL centrifuge tubes for the optimization of cell fragmentation conditions. Two distinct physical disruption techniques were employed to achieve cell lysis: high-pressure homogenization and ultrasonication. For the HPH method, we utilized the CF1 and CF2 Cell Disruptor (Constant Systems Ltd., Daventry, UK) and set the temperature to 5 °C while varying the crushing pressures to 10, 15, 20, 30, and 35 kpsi. In contrast, ultrasonication was performed using the Scientz-IID Ultrasonic Homogenizer (Ningbo Scientz Biotechnology Co., Ltd., Ningbo, China), with two variables considered: ultrasonic power and duration. The ultrasonic power settings were 100, 200, and 300 W, while the exposure times were 10, 20, and 30 min. To control temperature and prevent thermal degradation, samples were submerged in an ice bath throughout the ultrasonication process. A pulsed sonication protocol was applied, consisting of 5 s of ultrasonication followed by a 5 s interval, with this cycle repeated for the total ultrasonication time. Following cell disruption, protein concentration in each sample was quantified using the BCA Protein Concentration Determination Kit (P0012, Shanghai Beyotime Biological Technology Co., Ltd., Shanghai, China). The sample exhibiting the highest protein yield was designated as the reference sample, and all other samples were normalized to this standard to assess the efficiency of the cell-crushing methods.

### 2.5. Statistical Analysis

Each experimental group was replicated a minimum of three times, with triplicate measurements taken for each assay to enhance the reliability of the results. The graphical representations include error bars that represent the mean values accompanied by their standard errors of the mean (SEM). Statistical evaluations of the data were performed using one-way analysis of variance (ANOVA) and, where necessary, multivariate analysis of variance (MANOVA). Both the statistical computations and the creation of graphical representations were facilitated by the Origin 2023 software package (OriginLab Corporation, Northampton, MA, USA).

## 3. Results

### 3.1. Phylogenetic Tree and ITS2 Secondary Structure

**Figure 1:**
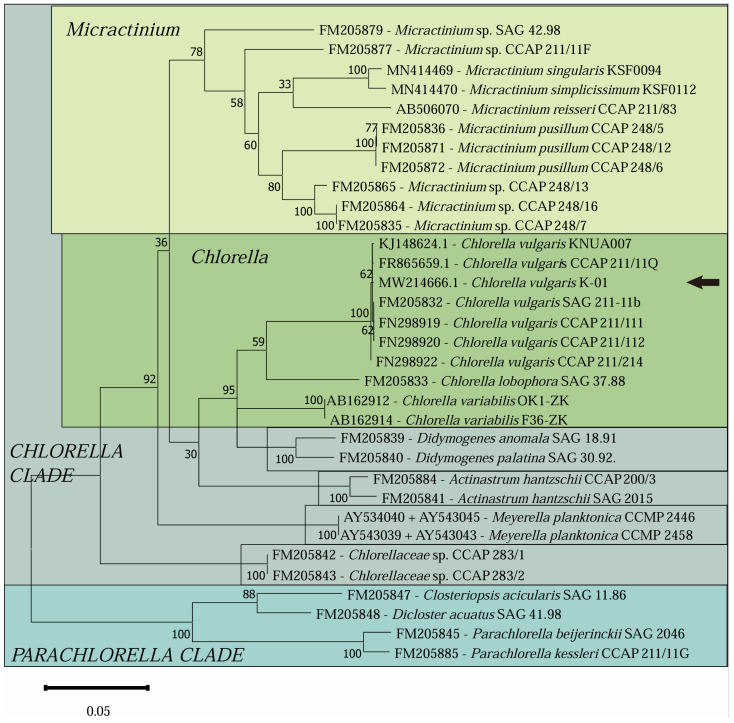
Maximum likelihood (ML) phylogenetic tree of *Chlorella vulgaris* K-01 constructed by SSU-ITS rDNA sequence alignments. The strain characterized in the current investigation is highlighted by a black arrow. This tree is scaled, with branch lengths representing the number of nucleotide substitutions per site. The numbers near the nodes are bootstrap values.

The compiled dataset encompassed 33 nucleotide sequences spanning 2475 nucleotide positions. The resulting ML phylogenetic tree is depicted in Figure 1. Our analysis revealed that the strain is part of the *Chlorella* clade, with *Chlorella vulgaris* KNUA007, *Chlorella vulgaris* CCAP211/11Q, and *Chlorella vulgaris* SAG 211-11b being the most closely related species.

**Figure 2:**
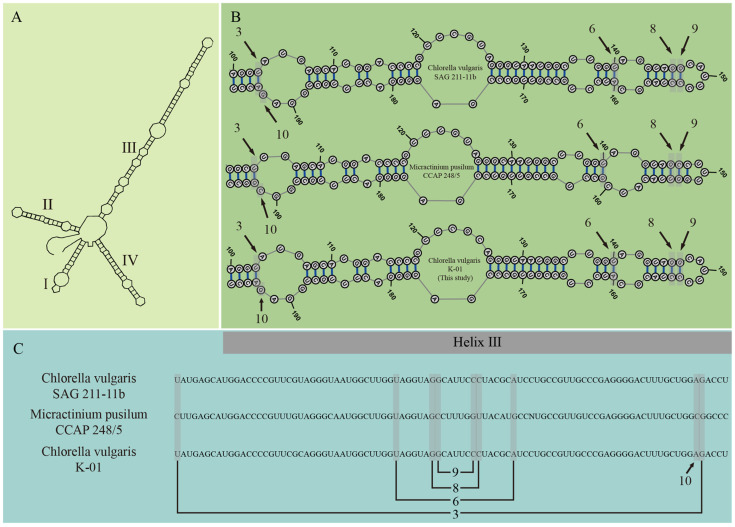
(**A**) Diagrammatic representation of the typical ITS2 secondary structure, with all helices designated by Roman numerals; (**B**) comparative analysis of Helix III within the ITS2 secondary structures for the verification of the isolated strain. The strain *Chlorella vulgaris* SAG 211-11b is utilized to depict the prototypical ITS2 structure characteristic of *Chlorella*, while *Micractinium pusillum* CCAP 248/5 is used to represent the canonical ITS2 structure of *Micractinium*. Distinctive features distinguishing *Chlorella* and *Micractinium* are annotated based on prior research by Luo et al. [56]; (**C**) linear sequence alignment of nucleobases within Helix III of the ITS2 secondary structures. The numbers annotated beneath the aligned sequences correspond to the specific positions on Helix III of the ITS2 secondary structures.

To discern between the morphologically similar and phylogenetically closely related genera *Micractinium* and *Chlorella*, we reconstructed the secondary structure of the ITS2 region. Figure 2A illustrates the overall ITS2 secondary structure, which features four helices, with an enlargement of the upper part of helix III to facilitate the comparison of ITS2 secondary structures and to confirm the clade of our isolate. Sequence alignment differences in secondary structures were also noted (Figure 2B), with corresponding numbers indicating the nucleobase positions on the secondary structure (Figure 2C). Specifically, position 9 is a characteristic synapomorphy for *Micractinium* [19,59]. At this position, *Chlorella vulgaris* species are expected to show a G–C base pair, as exemplified by the reference strain *Chlorella vulgaris* SAG 211-11b. In contrast, *Micractinium* species are expected to show a C–G base pair, as exemplified by the reference strain *Micractinium pusillum* CCAP 248/5. The presence of a C–G pairing at position 9 in the newly isolated strain confirmed it belongs to the *Chlorella vulgaris* and was named as *Chlorella vulgaris* K-01. The corresponding sequence of the strain was uploaded to GenBank (accession No. MW214666).

### 3.2. Measuring Parameters for PAM Fluorometry

**Figure 3:**
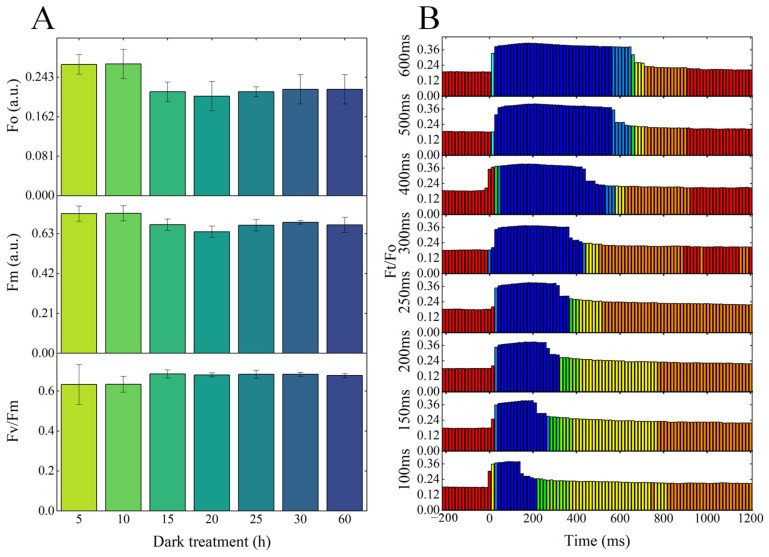
Optimization of dark treatment time and saturation pulse time for the determination of photosynthetic parameters of *Chlorella vulgaris* K-01. (**A**) Photosynthetic parameters of *Chlorella vulgaris* K-01 under different dark adaptation times; (**B**) chlorophyll-fluorescence response of *Chlorella vulgaris* K-01 at different saturation pulse times. The initial state of chlorophyll fluorescence, without saturation pulse excitation, is indicated by the red bars, while the excited state with saturation pulse excitation is denoted by the blue bars.

Variations in light response exist among different plant and microalgae species, which can impact the accuracy of photosynthetic response measurements. To minimize instrumental determination errors and obtain precise values for key photosynthetic parameters—namely, minimal fluorescence (Fo), maximal fluorescence (Fm), and the ratio of variable to maximum fluorescence (Fv/Fm)—we optimized the dark adaptation conditions for *Chlorella vulgaris* K-01. We set different dark adaptation periods of 5, 10, 15, 20, 25, 30, and 60 min and monitored the corresponding changes in Fo, Fm, and Fv/Fm, as depicted in Figure 3A. The Fo values for *Chlorella vulgaris* K-01 were 0.269, 0.27, 0.213, 0.204, 0.213, 0.218, and 0.204 a.u. at the respective dark adaptation times. The Fm values were 0.735, 0.737, 0.677, 0.64, 0.674, 0.69, and 0.676 a.u., while the Fv/Fm ratios were 0.633, 0.634, 0.686, 0.681, 0.684, 0.684, and 0.678, respectively. Our findings indicate that beyond 15 min of dark adaptation, Fo for *Chlorella vulgaris* K-01 decreased and stabilized around 0.215 a.u., Fm decreased and stabilized at approximately 0.67 a.u., and Fv/Fm increased and plateaued at approximately 0.68. Hence, 15 min of dark adaptation is enough for obtaining stable and accurate Fo, Fm, and Fv/Fm.

We further optimized the saturation pulse duration for *Chlorella vulgaris* K-01 by testing a range of pulse durations: 100, 150, 200, 250, 300, 400, 500, and 600 milliseconds. The outcomes of these pulse response tests are presented in Figure 3B. Initially, there was a sharp increase in chlorophyll fluorescence in response to the saturation pulse, reaching a peak value during the early phase of the pulse. When reaching this peak, the fluorescence response of the strain began to wane after it had been sustained for a certain duration. Towards the end of the saturation pulse, the fluorescence response plummeted, and then gradually returned to a stable state.

**Figure 4:**
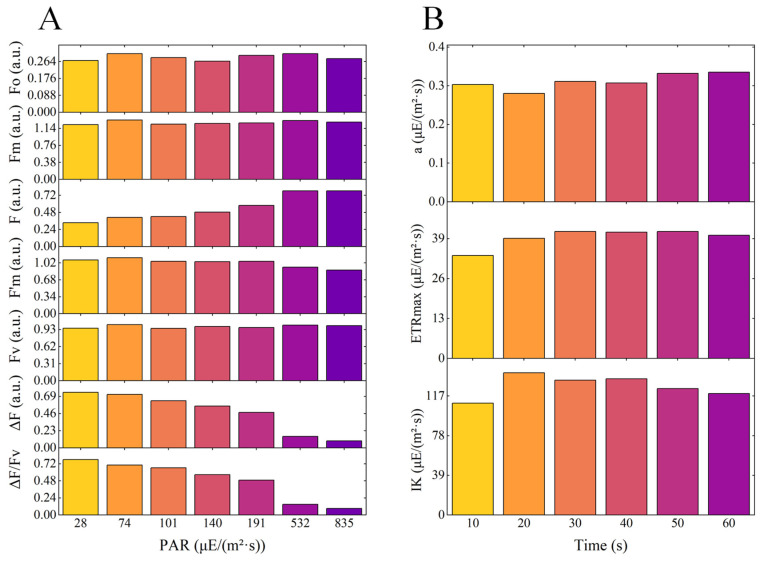
Optimization of the intensity (**A**) and the duration (**B**) of actinic light. α: initial slope of the RLC and ETR of the PS II (ETR (II) max) (μE/(m^2^·s)). ETRmax: maximum relative electron transport rate (μE/(m^2^·s)). Ik: light saturation point (μE/(m^2^·s)).

Figure 4A illustrates the photosynthetic response parameters, including initial fluorescence (F), maximal fluorescence (F’m), variable fluorescence (ΔF), and the efficiency of photosystem II photochemistry (ΔF/Fv) for *Chlorella vulgaris* K-01 under various actinic light intensities. These parameters were observed to vary with the intensity of the actinic light. Specifically, the ΔF/Fv ratio for *Chlorella vulgaris* K-01 decreased from 0.78 to 0.09 as the actinic light intensity increased from 28 to 835 μE/(m^2^·s). The empirical range for ΔF/Fv values, which is between 0.3 and 0.6 [60], was achieved at an actinic light intensity of 191 μE/(m^2^·s), establishing 191 μE/(m^2^·s) as the suitable actinic light intensity for evaluating the photosynthetic response parameters of this species. Figure 4B depicts the influence of varying actinic light exposure durations on the photosynthetic curve parameters of *Chlorella vulgaris* K-01. The parameters, including the initial slope (a), maximum electron transport rate (ETRmax), and light saturation point (IK), exhibited an initial increase followed by a stabilization as the actinic light exposure time was extended to 10, 20, 30, 40, 50, and 60 s. Notably, after 30 s of actinic light exposure, the photosynthetic curve parameters for *Chlorella vulgaris* K-01 entered a steady state. Consequently, an actinic light exposure time of 30 s was determined to be suitable for *Chlorella vulgaris* K-01, beyond which no significant changes in the photosynthetic parameters were observed.

### 3.3. Optimization of Microalgal Cell Fragmentation Conditions

**Figure 5:**
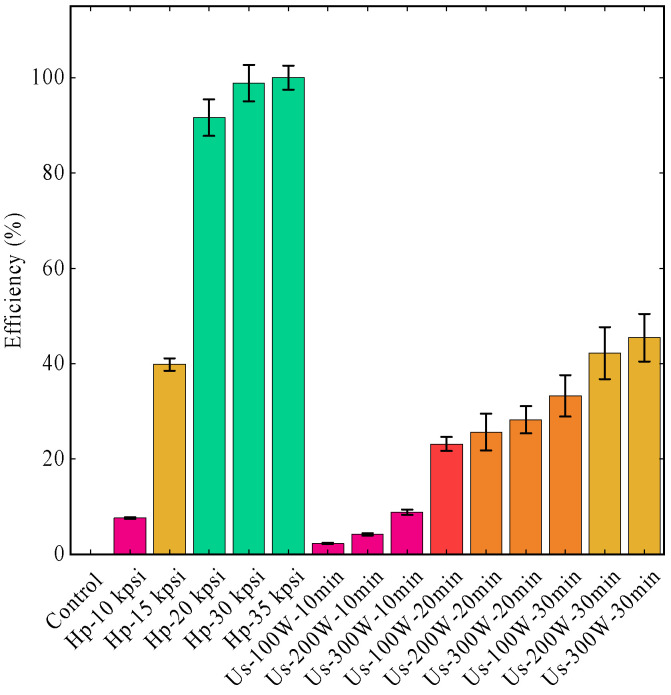
Cell disruption efficiency under different high pressure (Hp) or ultrasonic (Us) conditions.

The cell-crushing efficiency under high-pressure conditions was calibrated against a benchmark of 100% efficiency at 35 kpsi, and all subsequent data were normalized to this standard, with the results depicted in Figure 5. Under high-pressure cell crushing, the efficiency of cell disruption for *Chlorella vulgaris* K-01 progressively increased with escalating pressure, achieving 98.65% efficiency at 30 kpsi (207 MPa). Conversely, for ultrasonic cell crushing, the efficiency was observed to rise with both increasing ultrasonic power and duration, with an efficiency of 45.47% recorded at an ultrasonic power of 300 W and a duration of 30 min.

## 4. Discussion

In this paper, we present the identification of a distinct *Chlorella vulgaris* strain, designated as K-01, utilizing SSU-ITS phylogenetic analysis and ITS2 secondary structure assessment. Our study meticulously determined the suitable parameters for PAM fluorometry to accurately assess photosynthetic performance. Additionally, we conducted a thorough evaluation of various cell disruption techniques to enhance the extraction efficiency from microalgal cells.

Phylogenetic analysis is a critical tool in elucidating the taxonomic relationships and evolutionary trajectories of biological species. It is grounded in genetic principles, taxonomic classifications, and evolutionary biology, and it constructs a phylogenetic framework among species by analyzing DNA or protein sequence homologies. This approach is particularly valuable for classifying and identifying unknown or newly discovered species by referencing their sequence similarities to known species within the phylogenetic context [61]. The internal transcribed spacer 2 (ITS2) sequence, found within the ribosomal RNA of fungi, plants, and other eukaryotes, is renowned for its genomic variability, offering high discriminatory power at the species level. The secondary structure of ITS2 provides a wealth of information beyond the primary sequence, and it has become a pivotal tool in distinguishing between closely related species [18,62]. The genera *Chlorella* and *Micractinium*, which are morphologically similar and phylogenetically closely related, cannot be reliably differentiated based on morphology alone or on phylogenetic analysis of ribosomal small subunit sequences (SSUs) [56]. The comparison of ITS2 secondary structures enhances the precision of identification, particularly in differentiating between *Micractinium* and *Chlorella*, which are closely positioned on the phylogenetic tree [63]. Luo et al. [19] employed a combination of SSU-ITS sequence phylogeny and ITS2 secondary structure analysis to reclassify five algal species, initially identified as *Dictyosphaerium*, into *Chlorella* spp., demonstrating the enhanced accuracy of this integrated approach. Despite its proven utility, some recent studies on *Chlorella* isolation have overlooked this method, potentially leading to misidentifications and undermining the reliability of physiological test outcomes [64,65]. In our study, we adopted a dual approach of SSU-ITS rDNA sequence phylogenetic analysis and ITS2 secondary structure comparison to establish a robust molecular identification protocol for *Chlorella* species. This protocol aligns with methodologies used in previous algal classification revisions [19,66,67] and facilitates more precise classification of newly isolated *Chlorella* species, making it a recommended method for future research and exploitation of *Chlorella* resources.

PAM fluorometry is a widely recognized technique for probing the physiological conditions of microalgae, having been applied across diverse research areas. It is particularly instrumental in probing the physiological stress caused by nutrient fluctuations and the synthesis of cellular nutrients within *Chlorella* species [33], as well as in assessing the oxidative stress in *Chlorella vulgaris* triggered by exposure to hazardous substances [33]. However, the measurement conditions for PAM fluorometry, which are often derived from empirical observations, may lead to discrepancies between the measured values and the actual physiological metrics [44]. In the literature, the dark adaptation times reported for *Synechocystis* sp. PCC 6803 and *Chlorella sorokiniana* 211-8k are 30 min [44], while for *Dunaliella tertiolecta* CCAP 19/27, it is 10 min [68]. Our study determined that a 15 min dark adaptation period was adequate for achieving stable Fo, Fm, and Fv/Fm measurements in *Chlorella vulgaris* K01, with no substantial changes observed with further extension of the dark adaptation time. This finding suggests that the empirical dark adaptation times may not be appropriate for newly identified *Chlorella* species. Additionally, in this study, a reduced dark adaptation time could lead to a decrease in Fv/Fm, potentially leading to a misinterpretation that the test subjects are under physiological stress. These insights underscore the necessity for species-specific optimization of PAM fluorometry parameters to ensure the accuracy and reliability of physiological assessments.

For an accurate determination of photosynthetic parameters in species less studied, it is crucial that the chlorophyll response to the saturation pulse not only reaches its peak but also remains at that peak without a subsequent decline; otherwise, parameters such as Fv/Fm may not accurately reflect their true values [41]. Based on this, a saturation pulse duration of 200 milliseconds was deemed suitable for *Chlorella vulgaris* K-01. At this duration, the chlorophyll fluorescence of the strain reached a plateau without any subsequent decrease, ensuring the most precise measurement of its photosynthetic parameters.

In the realm of PAM fluorometry for assessing microalgal photosynthetic efficiency, the parameters of saturation pulse duration, actinic light intensity, and its duration have often been understated [69,70,71,72]. Our research has demonstrated that these factors significantly influence the precision of PAM fluorometry measurements. Consequently, it is imperative for forthcoming studies to meticulously calibrate these parameters prior to conducting device-based assessments. This attention to detail will enhance the reliability and validity of PAM fluorometry as a tool for microalgal analysis.

Microalgae exhibit variability in cell wall composition, which influences the efficiency of cell disruption methods. Tailoring the cell-crushing conditions is essential for the complete release of intracellular components such as nucleic acids, sugars, pigments, lipids, proteins, metabolites, and others, enabling subsequent physiological and functional studies of microalgae. In this investigation, *Chlorella vulgaris* K-01 served as the model organism to assess the efficiency of two cell-disruption techniques: high-pressure cell crushing and ultrasonic cell fragmentation. High-pressure homogenization (HPH) for cell lysis involves the use of elevated pressures to induce liquid shear stress and frictional forces to disrupt the cell walls previously described [73,74,75]. On the other hand, ultrasonication generates cavities in liquid, and they implode to release a significant amount of energy to dismantle the cell wall [76]. The efficacy of cell disruption techniques is significantly influenced by the microalgal species and the structure and composition of the cell wall, which in turn affects the efficiency of the extraction of intracellular compounds [74]. Microalgal cell walls can be categorized into two primary types based on their resistance: those with low lysis resistance and those with high lysis resistance, of which, microalgae species such as *Chlorella* and *Scenedesmus* are characterized by their highly lysis resistant cell walls [77,78]. Ultrasonication has been reported more effective for species with less lysis-resistant cell walls [79]. In the case of *Chlorella*, which possesses a robust cell wall, high-pressure homogenization was the most effective method, achieving 74% cell disruption, followed by chemical treatment at 33%, bead beating at 18%, and ultrasonication at only 5% [80]. In the context of our research, the application of ultrasonication for 30 min at a power level of 300 W resulted in a comparatively low microalgal cell lysis rate of 45.47%. This is significantly less than what was achieved with high-pressure cell disruption, which demonstrated an effectiveness of 98.67% in lysing microalgal cells at a pressure of 30 kpsi (207 MPa). The disparity in efficiency between these two methods suggests the superiority of high-pressure techniques in microalgal cell disruption.

## 5. Conclusions

In this research, we have successfully identified *Chlorella vulgaris* K-01 using SSU-ITS phylogenetic analysis and an assessment of the ITS2 secondary structure. The suitable parameters for the PAM fluorometry of *Chlorella vulgaris* K-01 were 15 min for dark adaptation, 200 milliseconds for saturation pulse, and 30 s for actinic light (191 μE/m^2^·s). The most suitable condition for *Chlorella vulgaris* K-01 cell disruption was high-pressure homogenization at 30 kpsi (207 MPa), obtaining 98.65% cell lysis efficiency. This foundational work paves the way for future comparative studies with other isolates and exploration into the potential of *Chlorella vulgaris* K-01 as a rich source of prebiotics and nutraceuticals. It is recommended that subsequent research focus on the extraction and characterization of bioactive compounds from this strain, which could contribute to human health and nutrition.

## Data Availability

The original contributions presented in this study are included in the article. Further inquiries can be directed to the corresponding authors.
